# One health – an ecological and evolutionary framework for tackling Neglected Zoonotic Diseases

**DOI:** 10.1111/eva.12341

**Published:** 2016-01-08

**Authors:** Joanne P. Webster, Charlotte M. Gower, Sarah C. L. Knowles, David H. Molyneux, Andy Fenton

**Affiliations:** ^1^ Department of Pathology and Pathogen BiologyCentre for Emerging, Endemic and Exotic Diseases (CEEED)Royal Veterinary CollegeUniversity of LondonHertfordshireUK; ^2^ Department of Life SciencesImperial College LondonAscotBerkshireUK; ^3^ Department of ParasitologyLiverpool School of Tropical MedicineLiverpoolUK; ^4^ Institute of Integrative BiologyUniversity of LiverpoolLiverpoolUK

**Keywords:** disease control, ecology, evolution, key hosts, NTDs, NZDs, preventive chemotherapy, zoonoses

## Abstract

Understanding the complex population biology and transmission ecology of multihost parasites has been declared as one of the major challenges of biomedical sciences for the 21st century and the Neglected Zoonotic Diseases (NZDs) are perhaps the most neglected of all the Neglected Tropical Diseases (NTDs). Here we consider how multihost parasite transmission and evolutionary dynamics may affect the success of human and animal disease control programmes, particularly neglected diseases of the developing world. We review the different types of zoonotic interactions that occur, both ecological and evolutionary, their potential relevance for current human control activities, and make suggestions for the development of an empirical evidence base and theoretical framework to better understand and predict the outcome of such interactions. In particular, we consider whether preventive chemotherapy, the current mainstay of NTD control, can be successful without a One Health approach. Transmission within and between animal reservoirs and humans can have important ecological and evolutionary consequences, driving the evolution and establishment of drug resistance, as well as providing selective pressures for spill‐over, host switching, hybridizations and introgressions between animal and human parasites. Our aim here is to highlight the importance of both elucidating disease ecology, including identifying key hosts and tailoring control effort accordingly, and understanding parasite evolution, such as precisely how infectious agents may respond and adapt to anthropogenic change. Both elements are essential if we are to alleviate disease risks from NZDs in humans, domestic animals and wildlife.

## Introduction

At the beginning of the twenty‐first century, the world is faced with a changing landscape of infectious diseases that affect both humans and animals, many of which can pose significant threats to health and welfare. Threats from old and new parasites and pathogens continue to emerge, fuelled by changes in the environment (climate, dam constructions, deforestation, disruption of ecosystems, etc.), in agriculture and food production (from intensive systems of husbandry and farming monoculture to changing of traditional patterns of livestock movements), and in the demography and connectivity of the modern ‘global’ village (population growth, urbanization, international trading, world tourism and rapid transportation) (Gibbs 2005; Johnson et al. 2015). Anthropogenic changes, particularly those involving movements of infected people and animals or that change habitats in a manner likely to provide new opportunities for host‐parasite mixing, can further drive the introduction of both known and novel parasite genotypes to previously unaffected host individuals or species. In recent years, the emergence or re‐emergence of animal and human infectious diseases has been increasingly documented around the world (Woolhouse et al. 2005), with an average of three new human infectious diseases being reported approximately every two years, and a new infecting organism described every week (http:/ www.gideononline.com) (Tomley and Shirley 2009). Ancient diseases, such as schistosomiasis (Berry et al. 2014; ECDC 2014; Laval et al. 2014; Boissier et al. 2015), are also presenting in novel environments and causing new or changing patterns of disease as human populations and their environments grow and change. Attempts to control infectious agents through large‐scale drug distribution or vaccination also add to the ever‐changing environment in which parasites and pathogens must either evolve, adapt or succumb (Webster et al. 2008, 2014).

At least 60% of human diseases and 60–75% of new emerging diseases are multihost zoonoses (Cleaveland et al. 2001). Zoonotic reservoirs can maintain infections in times of change, thwart attempts to control or eliminate disease in human populations, as well as influence parasite evolution by providing opportunities for host switching or genetic exchange giving rise to novel genetic combinations. The World Bank has estimated that zoonoses have cost global economies more than $20BN in direct, and $200BN in indirect, costs between 2000 and 2010. Zoonotic disease risks are predicted to further increase as environmental change continues. The global human population is expected to increase from approximately 7.2 billion in 2014 to approximately 9.2 billion by 2050, with around one billion of this increase occurring in Africa alone (UNDP 2008). Standing populations of livestock in 2007 were estimated at 1.43 billion cattle, 1.87 billion sheep and goats, 0.98 billion pigs and 19.6 billion chickens, with average yearly increases of 5.1% and 3.6% in developing country meat and dairy sectors, respectively, since 1970 (WHO 2012a; Robinson et al. 2014).

The ‘megacities’ of the world constitute obvious melting pots for the mixing of human and animal parasites and their potential rapid spread, both locally and internationally. For instance, although transmission of SIV from chimpanzees to humans may have occurred on a number of distinct occasions (Hahn et al. 2000), such spill‐overs (Table 1) remained isolated and only through urbanization and increased global travel did the HIV pandemic take off in the 20th century (Fenton and Pedersen 2005). Urbanization and large‐scale human population movements, often associated with conflict, have similarly been implicated in more recent outbreaks of potentially zoonotic origin diseases from leishmaniasis (Saroufim et al. 2014) to Ebola (Faye et al. 2015). However, rural landscapes also pose ever increasing risks for the mixing of human and animal parasites, especially those exposed to recent anthropogenic changes such as new dam construction, flooding and changes in animal husbandry. The Food and Agriculture Organization estimates that livestock contributes to the livelihoods of 70% of the world's rural poor (WHO 2006). Not only are poor people and their livestock more at risk of contracting a range of zoonoses, once infected, it is the poor that are least likely to have access to health services and hence get appropriate medical or veterinary care.

**Table 1:** Human‐targeted versus Animal‐targeted control measures for some of the major NZDs and NTDs

Disease	Infectious agents	Known Animal Host Reservoirs	Current Human‐ focused control	Current Animal‐focused control
Schistosomiasis	Urogenital form: *Schistosoma haematobium* Intestinal from: *S. mansoni, Schistosoma japonicum*,* S. mekongi* or *S. intercalatum*.	*S. japonicum*: Large number of domestic and wildlife reservoirs – in particular bovines and rodents, respectively; *S. mansoni*: baboons, rats *S. haematobium*: was assumed human‐specific, but *S. haematobium*‐group introgressed hybrids suspected within livestock and potentially wildlife, currently under investigation	Preventive chemotherapy with praziquantel across parts of sub‐Saharan Africa, Asia, the Arabian Penisular, and South America Focal mollusciciding in some settings, for example China and Zanzibar	Preventative chemotherapy with praziquantel and vaccine under development for *S. japonicum* in bovines only in China. Elsewhere no animal‐focused control
Soil‐transmitted helminthiasis	*Ascaris lumbricoides* *Necator americanus* *Ancylostoma duodenale* *A.ceylanicum* *Trichuris trichuria*	Pigs Dogs, cats, wild canids Baboons; pigs? Dogs?	Preventive chemotherapy with albendazole or mebendazole Ivermectin used for onchocerciasis control will also impact on STH	Regular de‐worming of pet dogs and cats in developed countries; No organized control in developing countries
Lymphatic filariasis	*Wuchereria bancrofti* *Brugia malayi/B. timori*	Cats and leaf monkeys *(Presbytis*) can be infected with *Brugia malayi* although significance unknown No known animal reservoirs for *W. bancrofti*	Annual Preventive chemotherapy with Ivermectin and albendazole or albendazole with Diethylcarbamizine Vector control in *Anopheles* transmitting settings in Africa and Papua New Guinea	No
Onchocerciasis	*Onchocerca volvulus*	None known, although related species in cattle, for example *O. gutturosa*	Preventive chemotherapy with Ivermectin	No (but no animal hosts confirmed)
Trachoma	*Chlamydia trachomitis*	None known, although closely related species in animals; thought to be separate species	Preventive chemotherapy with azithromycin	No (but no animal hosts confirmed)
Dengue fever	Dengue virus	Primates	Vector control	No
Chagas disease	*Trypanosoma cruzi*	Domestic transmission cycles: dogs, cats Sylvatic transmission cycles: over 180 small mammal species particularly rodents, bats and Opossums (peri‐domestic reservoir) Domestic & sylvatic cycles overlap in some regions	Vector control	No
Human African Trypanosomiasis	*Trypaonsoma brucei gambiense* *T.b. rhodisiense*	Pigs, cattle, squirrels, porcupines, monkeys potentially pigs. Wild ungulates, cattle,	Active surveillance to detect cases and treat; vector control Case finding and treatment	Not for *T. b. gambiense* Chemotherapy of cattle reservoir (Uganda) accompanied by selective spraying of insecticides on cattle
Leishmaniaisis	*Leishmania infantum, L.donovani* (viscercal leishmaniasis) *Up to 15 Leishmania* spp. *inc. L. major & L. tropica* (cutaneous leishmaniasis)	Dogs No known reservoir of *L.donovani* in Asia Rodents (Africa & EurAsia & Americas)	Improved diagnostic methods for case identification aim to increase access to drugs and decrease drug prices. Case detection through improved diagnostics. Vector control through indoor residual spraying	Some case detection in dogs in developed countries. Use of insecticide impregnated collars or pour –on insecticides for treatment of dog reservoirs For *L. major* in North Africa, Middle east/ Central Asia destruction of rodent habitats and vegetation on which reservoir rodents dependent
Rabies	Rabies virus (a lassa virus)	Dogs and wild canids, cats, horses, mongooses, primates, racoons, sheep, skunks and wolves.	Health education. Availability of postexposure prophylaxis vaccines	Systematic culling of feral and domestic dogs in some regions (e.g. Philippines) Mass vaccination of dogs, for example South America, KwaZulu‐Natal province of South, Bali, Indonesia
Cystercercosis taeniasis	*Taenia solium*	Pigs	Individual treatment with praziquantel – Plans for preventive chemotherapy in progress	Meat inspection; health education Treatment and/or vaccination of pigs in planning stages
Guinea worm ‐ Dracunculiasis	*Dracunculus medicinsis*	Dogs and potentially also cats	Temephos treatment of water bodies to kill copepod intermediate hosts; filtering of potentially contaminated water; provision of safe drinking water sources; case containment of new cases to prevent access to water bodies	Case detection of dogs in Chad recently initiated; reward for reporting infected dogs; tethering dogs to prevent access to water bodies
Food‐borne trematodiasis	*Paragonimus* spp. *Opisthorchis viverinni O. felineus*. *Clonorchis spp*. *Fasciola hepatica and F. gigantica*	Dogs, cats, wildlife Cats, dogs, wildlife Cats, dogs, foxes, pigs Cats, dogs, fish‐eating mammals Cattle, sheep, buffalo, donkeys, pigs, other herbivores	‒ Individual treatment with PZQ or triclabendazole Mass screening in some areas	No
Buruli ulcer	*Mycobacterium ulcerans*	Unknown	Early diagnosis and antibiotic treatment Surgery of advanced lesions	No (but no animal hosts confirmed)
Leprosy	*Mycobaterium leprae*	Armadillos, nonhuman primates	Multidrug therapy available free of charge from WHO for all endemic countries	No
Yaws	*Treponema pallidum*	None known‐ but *Treponema* sp. present in nonhuman primates	Antibiotic treatment	No (but no animal hosts confirmed)
Anthrax	*Bacillus anthracis*	Domestic and wild herbivores	Vaccination and antibiotic treatment	Vaccination and antibiotic treatment
Bovine TB	*Mycobaterium bovis*	Wide range of mammalian hosts	BCG vaccination in some developed countries	Systematic culling of domestic and wildlife in developed countries No or limited control in developing countries
Brucellosis	*Brucella sp*.	Domestic animals esp cattle & goats, wildlife, dogs	Health education on risk of contact with livestock/products	Vaccination of cattle in developed countries
Cystic echinococcus Alveolar echinococcosis	*Echinococcus granulosus* *E. multilocularis*	Dog, sheep, goats, camels, yak, cattle camels Rodent reservoirs	Health Education on dog role and slaughter of sheep	Vaccination of lambs promising. Deworming of dogs Appropriate disposal of offal containing cystic stages and preventing access of carnivores to cystic stages

The Neglected Tropical Diseases (NTDs) are highly debilitating diseases caused by a range of viruses, bacteria, protozoa and helminths which infect more than one billion people, a sixth of the world's population, but predominantly the poorest of the poor (Hotez et al. 2009). An initial assessment suggested that 14 of the major NTDs kill an estimated 534 000 people worldwide every year, while causing a disease burden measured in Disability‐Adjusted Life Years (DALYs) that competed with HIV/AIDS, tuberculosis and malaria (Hotez et al. 2006). However, as the name suggests, NTDs have been identified as diseases that receive less attention and funding compared to those other diseases (Liese et al. 2014). We are, however, currently in an exciting era of disease control, at least with respect to several of the human NTDs (Webster et al. 2014). A subset of seven NTDs are being targeted with preventive chemotherapy through Mass Drug Administration of inexpensive, effective, oral drugs in affected communities (Table 1). The term ‘preventive chemotherapy’ was introduced by the WHO to define the strategic approach of treating populations infected, or at risk of being infected, with these NTDs without the need for individual diagnosis (WHO 2006). Some of these diseases have already been targeted for elimination (e.g. lymphatic filariasis, onchocerciasis and trachoma), while successful morbidity control against others (e.g. schistosomiasis and soil‐transmitted helminthiasis (STH)) has recently also shifted the current agenda towards ‘elimination as a public health problem by 2020’ (WHO 2012b; Webster et al. 2014).

Neglected Zoonotic Diseases (NZDs) are a critically important subset of the NTDs. Zoonoses are diseases that are naturally transmitted between vertebrate animals and humans, a process that increases the potential complexity of developing sustained and effective control, and demands a One Health perspective. While control programmes to date tend to be human focused only (notwithstanding the use of vector control in certain settings), the WHO Department of Control of NTDs has identified rabies, taeniasis/cysticercosis, zoonotic human African trypansomiasis, echinococcosis and food‐borne trematodiases as priority NZDs, with the potential to target control at both human and animal hosts (Table 1). Moreover, many of the drugs used in human treatment programmes are also active against nontarget parasite species, some of which infect animals. For example, praziquantel, used in human schistosomiasis control, is also active against the food‐borne trematodes paragonimiasis, opithorchiasis and fasciololaiasis, as well as echinococcus and taenia/cysticercosis, all of which are zoonotic (Table 1). In fact, many, if not all, of the drugs currently used to treat human NTDs are the same as those used treat animal diseases and indeed were initially developed for veterinary use (Table 1). The use of these drugs in humans may therefore also affect parasites in animal populations, with potential feedbacks to human disease through zoonotic transmission. The broader scale community dynamics, in terms of selective pressures placed on the infectious agents, though rarely studied, could have profound impacts on their potential for evolution and spread. For instance, mismanagement of anthelminthics in animals increases the risk of drug resistance evolving, thereby making drugs against zoonotic diseases no longer available to humans.

We are also in an exciting time in terms of improved molecular and diagnostic tools. Genetic and genomic data have become increasingly important in identifying and characterizing zoonotic parasites, and a proliferation of cross‐host species interactions have recently been revealed, which may have substantial implications from both epidemiological and evolutionary perspectives (King et al. 2015; Lamberton et al. 2015). Analysis of molecular sequence data and population genetics also offers unprecedented opportunities to understand micro‐evolutionary processes and the response to selection of such infections, as well as to elucidate relationships and genetic exchange between the parasites that infect different host species. For example, analyses of supposed ‘human‐specific’ and ‘animal‐specific’ species have revealed instances of ‘cryptic’ or ‘covert host specificity’, where parasites previously characterized as generalists (capable of infecting many host species), have turned out to rely heavily on single‐host species, and populations of relatively host‐specific strains circulate independently in different host species. Examples of presumed generalist pathogens that have since been shown to have cryptic host specificity include rabies virus in bats (Streicker et al. 2010) and parasitoid flies of insects (Smith et al. 2006). Conversely, molecular sequencing has now also revealed several instances of ‘covert generalists’, where parasites thought to be different because they were initially diagnosed in different host species turn out to be the same. For example, there are the morphologically similar species of *Ascaris* nematodes in pigs *(A. suis)* and humans (*A. lumbricoides)*, which occur sympatrically in many foci. Betson and colleagues (Betson et al. 2014) conducted a molecular study of *Ascaris* in pigs and humans from across Europe, Africa, Asia and Latin America and found that while there was indeed marked genetic segregation between worms originating from human and pig hosts, human *Ascaris* infections in Europe were found to be of pig origin and there was evidence of cross‐transmission between humans and pigs in Africa (Betson et al. 2014). The authors thus concluded that cross‐transmission of *Ascaris* between humans and pigs can occur in both developing and developed countries, with differing zoonotic interactions contingent upon epidemiological potential and local phylogeography. Similarly, there has been a continuing controversy about the taxonomic status of *Trichuris suis* from pigs and *Trichuris trichuria* in humans, and whether pigs are a zoonotic source of human infection. Cutillas et al. (2009) concluded that while they are separate parasite species based on ITS1 & ITS2 sequences, cross‐infection between host species do occur.

**Figure 1:**
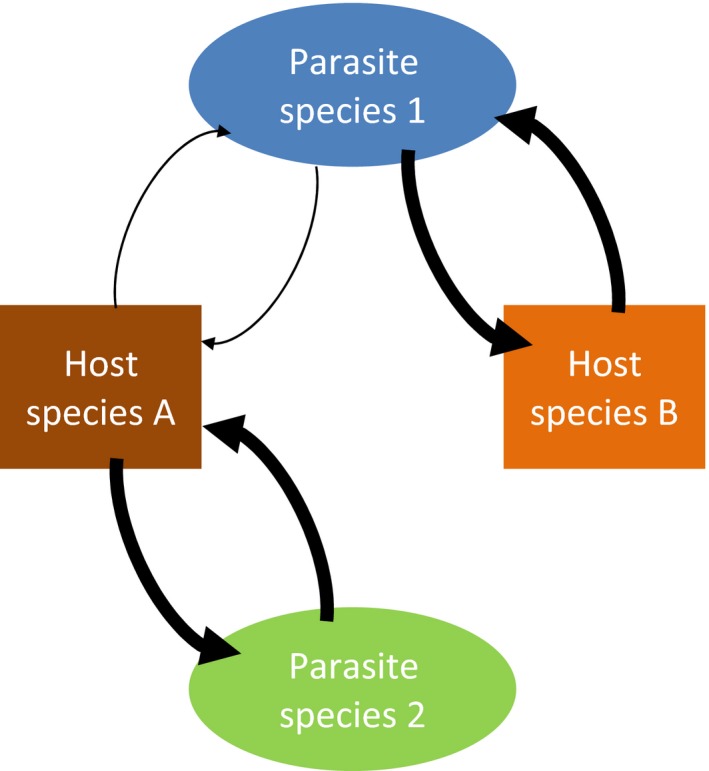
Schematic representation of a general multihost – multiparasite scenario. Parasite species 1 is the focal parasite of interest, and is a generalist (see Box 1 glossary) able to infect both host species A and B. However, host species B is the key host species for this parasite, dominating transmission. In the extreme, if host species B is a maintenance host and species A is not then, in the absence of any evolutionary response by the parasite, treating host species B will result in elimination of parasite species 1. However, parasite 1 may be able to evolve in response to that treatment, either by evolving resistance to the treatment, or undergoing a host shift to be maintained on host species A. In the scenario shown this would then expose it to possible co‐infections with parasite species 2. Parasite species 2 could act to facilitate or suppress the likelihood of species 1 establishing in the new host and, if the two parasite species are sufficiently related, could result in hybridization and/or introgression between them.

Understanding the complex population biology and transmission ecology of multihost parasites has been declared as one of the highest priorities for biomedical sciences for the 21st century (Woolhouse and Dye 2001; Woolhouse et al. 2001, 2002) and multihost zoonotic diseases are perhaps the most neglected of all the neglected diseases (Webster et al. 2014). Furthermore, seldom are the potential evolutionary responses by parasites considered within control programmes. Such responses may be exacerbated when we recognize that focal parasite and host species are embedded within a wider community of host and parasite species, which may greatly alter parasite gene flow through the community, enhance or diminish selection pressures imposed on parasites, and even result in the emergence of novel hybrid parasite species (Fig. 1). Clearly a more complete, One Health perspective, of the ecological and evolutionary settings of multihost zoonotic diseases is needed if we are to develop effective, sustained disease management strategies for these neglected diseases. Here, we address these issues by reviewing the different types of zoonotic interactions that may occur, the potential evolutionary impact of current human control activities such as preventive chemotherapy, and make suggestions for the development of an empirical evidence base and theoretical framework to better understand and predict the likelihood and outcome of such interactions.

Box 1 **Glossary of Transmission Terms**

**Table:** 

Term	Definition
Apparent Multihost Parasite	Infections occurring in more than one host species that arise from frequent cross‐species transmission from a maintenance host species or population to a nonmaintenance host species or population (Fenton and Pedersen 2005). Due to the high frequency of cross‐species transmission, infections in the nonmaintenance host are continuously observed, unlike in the case of ‘spill‐over’ infections. As with spill‐over infections though, removal of the maintenance host, or blocking of cross‐species transmission, would cause infections in the nonmaintenance host species to fade out.
Critical Community Size (CCS)	The minimum threshold population size for a host species to maintain parasite infection; only those host species with a population size in excess of the CSS will be capable of independent sustainment of transmission in the absence of other host species (Viana et al. 2014). Maintenance hosts, by definition, have population sizes in excess of the CSS, whereas nonmaintenance hosts do not.
Cryptic host specificity	Where apparently generalist parasites, which appear to infect multiple host species, actually comprise distinct subspecies or strains that circulate within each host species independently of each other. Cryptic host specificity is increasingly being revealed through increasing use of genetic sequencing.
Dead End Hosts	Host species that can become infected by a given parasite, but are not able to transmit those infections onwards to other individuals.
Generalist parasite	In the broadest definition, a parasite that is capable of infecting more than one host species, whether or not it is able to transmit onwards from, or be maintained by, each host species. There may be different types of generalist parasites, depending on their ability to transmit onwards from the different host species (if they cannot, then those species are deemed to be dead end hosts), or be maintained by those different host species in the absence of transmission from other host species (i.e. maintenance host species). Generalist parasites that have multiple maintenance host species are deemed to be true multi‐host parasites.
Host switching or Host shift	Where a parasite jumps from one host species to another. Host shifts appear to be common, with the phylogenies of hosts and parasites often showing incongruence, suggesting parasites have switched between host species. Past host switches within a group of parasites are often inferred from a comparison of the parasite phylogeny with that of the hosts. Congruence between the phylogenies is often attributed to a history of association by descent with co‐speciation, and incongruence to host switching or extinction in ‘duplicated’ parasite lineages, which diverged without a corresponding branching of the host tree.
Hybridization	From a taxonomic perspective, hybrid refers to offspring resulting from the interbreeding between two animal species or plant species – usually between species in the same Genera. An intraspecific hybrid may refer to crosses between subspecies or different populations of the same species.
Introgressive hybridization	Introgression, also known as introgressive hybridization, in genetics it is the movement of a gene (gene flow) from one species into the gene pool of another by the repeated backcrossing of an interspecific hybrid with one of its parent species. Introgression is an important source of genetic variation in natural populations and may contribute to adaptation and even adaptive radiation. Introgression differs from simple hybridization. Introgression results in a complex mixture of parental genes, while simple hybridization results in a more uniform mixture, which in the first generation will be an even mix of two parental species.
Key host	From ‘keystone’ ‒ something on which other things depend on, in this case the infectious agent. Even the most generalist of parasites often predominantly infect, and transmit from, a subset of potential hosts. Host species (where host here also included intermediate hosts and/or vector species (Molyneux 2003)) that individually contribute significantly to long‐term parasite persistence, and drive infection risk in sympatric host species relative to other host species, may be deemed to be key hosts. Key hosts can arise through different mechanisms (i.e. super‐abundant, super‐infected or super‐shedding, which may be due to innate differences among the species (i.e. genetic compatibility), co‐infection by other parasite species facilitating infection and transmission by the focal parasite, or may even arise through behavioural modification by the parasite to facilitate super‐infectivity. These different types of key host can have important implications for the optimal targeting of control. Hence, not only identifying key host species, but identifying which kind of key host species they are, is imperative for optimal targeting of control strategies.
Maintenance host	A population (or community) that is capable of sustaining a parasite, independent of epidemiological input from elsewhere (Haydon et al. 2002). A maintenance host population has a population size in excess of the Critical Community Size (CSS), the minimum density required to sustain the parasite (Viana et al. 2014). In deterministic models this equates to the basic reproduction number *R*_*0,i*_ exceeding 1, although this criterion is not always sufficient for parasite persistence where, for example, parasites with *R*_*0*_ > 1 may be lost through stochastic fade out (Lloyd‐Smith et al. 2005). A nonmaintenance host population is unable to maintain the parasite in the absence of external input, and so has a population size below the CSS.
Paratenic Host	A host that is not necessary for the development of a particular species of parasite or pathogen, but can serve to maintain the life cycle of that parasite/pathogen. In contrast to its development in an intermediate, secondary or definitive host, a parasite in a paratenic host does not undergo any subsequent changes in its development.
Potential Emerging Infectious Disease	A parasite that would be capable of infecting a novel host species, but is prevented from doing so purely through an ecological or host‐behavioural barrier to exposure (Fenton and Pedersen 2005). If that barrier is breached (for example, through anthropogenic change that facilitates contact between species), the infection will take off in the previously naïve host population.
*Refugia*	In parasitology *Refugia* refers to the parasite population that have not been exposed to a particular drug and hence still contains a large proportion of susceptible organisms. Having a large *Refugia* lessens the selective pressures placed on the parasite for the evolution of drug resistance. *Refugia* in terms of mass drug administration may relate to the human populations not treated, but also in terms of animal reservoirs of infection not exposed to drug treatment programmes.
Reservoir Host	One (or more) host populations in which a parasite can persist, and which acts as a source of infection to a target host species (Haydon et al. 2002). Some reservoirs can be simple and comprise a single nontarget host population. However, reservoirs of infection can be ecologically complicated structures comprising one or more interacting host species in which a parasite can be permanently maintained and from which infection is transmitted to the target host population.
*R*_*0*_, the basic reproduction number	A parasite's fitness can be measured by its basic reproduction number, or *R*_*0*_, which is defined as the number of new infections (for a microparasite, such as a virus, bacteria or protozoan) or new adult parasites (for a macroparasite, such as a helminth or ectoparasite) arising from a primary infection in a wholly susceptible host population or community. This definition provides a threshold for parasite invasion into a naïve host population; if *R*_*0*_ > 1, then the parasite can invade, if not then the parasite cannot. In deterministic models this also equates to the condition for parasite persistence within that host population. In a multi‐host species context, the overall *R*_*0*_ of the parasite within the community (*R*_0,TOT_) depends on the combined contributions of each of the *i* host species; *R*_*0,i*_ (Fenton et al. 2015; Dobson 2004).
Spill‐over Host	A nonmaintenance host population which encounters occasional cross‐species transmission from a maintenance host population or reservoir. Unlike apparent multi‐host parasitism, spill‐overs cause transient infections in the spill‐over (nonmaintaining) host population. Removal of the maintenance host, or blocking of cross‐species transmission, would cause infections in the nonmaintenance host species to fade out.
Target host or Target host population	The study of epidemiology is often motivated by the need to control disease in a particulate host population (e.g. a human population) or subset of a population. This can be referred to as the target host or target host population that may be risk of infection from a Reservoir or Source population.(Haydon et al. 2002; Viana et al. 2014); it may or may not be a maintenance host population in its own right.
True Multihost Parasite	Infections in multiple host species that are able to be maintained by those species in the absence of any other host species. Cross‐species transmission may occur, but is not essential for parasite maintenance in either host species. Removal of either host species, or blocking of cross‐species transmission, would not lead to loss of infection from the other host species, although prevalence may decline if rates of cross‐species transmission were significant (Fenton and Pedersen 2005).

## Understanding zoonotic disease transmission: the need to identify and characterize ‘key hosts’

While much epidemiological theory uses a single‐host–single‐parasite framework, in reality most diseases globally, including emerging and re‐emerging diseases in humans, involve multiple hosts (Cleaveland et al. 2001; Pedersen and Davies 2009). Animal reservoirs can maintain transmission with zoonotic parasites, even while the disease they cause in humans is effectively controlled (Box 1; Table 1). This is common and not restricted to tropical diseases, with examples such as the maintenance of bovine TB by reservoir hosts including, but not necessarily exclusive to, badgers in the UK (Mathews et al. 2006). The epidemiology of multihost parasites can involve a complex community of reservoir and vector species, and where infected hosts may range from spill‐over to maintenance hosts and from relative specialist parasites to true multihost generalists (Box 1). Within this context, the importance of differing hosts may vary and it is imperative, if often difficult, to identify which is which. Each host species is unlikely to contribute equally to parasite transmission, as they differ in abundance, exposure and susceptibility, and the transmission pathways among them (Haydon et al. 2002; Fenton et al. 2015). Even parasites with a very broad host range often occur or are transmitted predominantly by just a subset of potential hosts, or ‘key host’ species (Box 1). Moreover, for a given zoonotic parasite, the relative importance of different host species, that is the key hosts, can vary across ecological settings. These host species (which can also refer to intermediate host and vector species (Molyneux 2003)) not only act as a source of parasitic infections but also play a fundamental role in driving parasite gene flow through the community. Therefore, they can act as a driver of evolutionary change, but also potentially be a source of genetic bottlenecking within the parasite population. Due to both the clear importance of understanding such heterogeneities in disease transmission across host communities (for instance, for effective control, we would need to identify which species are important for amplifying transmission and transmitting to target host populations), and the likelihood that evolutionary selective forces may differ between such hosts and environments, it is thereby imperative to first identify key host species acting as reservoirs of infection (Haydon et al. 2002). However, to fully understand and predict the transmission dynamics of any infection, it is also essential to know the relative contribution of any particular host species to the transmission dynamics of the parasite (Rudge et al. 2013; Fenton et al. 2015) and the importance of the host species to the evolutionary pressures acting on the parasite. At the extreme, multihost parasites may depend on a single species for long‐term persistence, a ‘maintenance’ key host (Box 1). Being able to identify these key hosts is essential in determining which host species to target for disease control (Haydon et al. 2002; Fenton and Pedersen 2005). For example, rabies virus infections in wild carnivores in the Serengeti depends on viral maintenance by domestic dogs, so vaccinating dogs is expected to alleviate epizootics in wildlife (Lembo et al. 2008). Consequently, interventions such as vaccination, culling or sterilization commonly target single species rather than all infected hosts. However, it is also important to consider the distinct processes by which host species contribute differentially to parasite transmission and maintenance, and the different ways in which key hosts can arise, a feature that has received relatively little attention (Streicker et al. 2013). A host species can make disproportionate contributions to the total infectious pool by being, for instance, ‘super‐abundant,’ ‘super‐susceptible’, ‘super‐infected’ and/or a ‘super‐shedder’. That is, the relative contribution of any individual host species to a parasite's total transmission pool is proportional to the product of that species’ abundance, infection and shedding (Streicker et al. 2013). For instance, the importance of dogs for rabies transmission is driven at least partly by their higher population densities relative to other carnivore species (Lembo et al. 2008), but West Nile virus transmission around Washington D.C. is dominated by the presence of a relatively rare, but highly infectious bird, the American robin (Kilpatrick et al. 2006). Furthermore, certain host species may have behavioural patterns that make them key hosts, where even if not, for instance, highly abundant, their behavioural repertoires place them in high contact with humans and/or other suitable host species. There are numerous cases of such behavioural alternations and modifications within parasitized invertebrate, including vector, host species, although vertebrate host cases are rarer (Adamo & Webster, 2013). One example may be the roosting behaviour and habitat selection of bats and their link to Nipah virus epidemiology (Hahn et al. 2014). Even more intriguing are cases where certain indirectly transmitted parasites can adaptively manipulate host behaviour in a manner to enhance transmission. Prime examples of such manipulation include the increased aggression proposed to enhance transmission via biting of viruses such as rabies, Hantaan and Seoul viruses through blood and saliva (Kaushik et al. 2012) to the subtle ‘mind’ alteration of infected rodents by *Toxoplasma gondii*, that enhances predation by its feline definitive host and therefore transmission (Webster 2007; Webster et al. 2013b; Kaushik et al. 2014).

While for many NZDs it remains poorly understood how different host species contribute to transmission and human infection, studies of schistosomiasis in Asia illustrate how knowledge about key hosts can inform disease control. Both *Schistosoma japonicum* and, to a lesser extent *S. mekongi*, which occur in Asia (China, The Philippines, and parts of Laos and Cambodia) are zoonotic, where water buffalos and other bovines were considered the major definitive hosts. In China, for example, after five decades of concerted and multifaceted interventions (including chemotherapy, mollusciciding, health education, sanitation and environmental improvement), *S. japonicum* remains endemic among humans (Zhou et al. 2007) and has even re‐emerged in areas where it was thought to have been eliminated (Liang et al. 2006). Novel analyses involving a combination of parasitological field data, population genetics and mathematical modelling using a framework that partitioned the contributions different host species made to the parasites basic reproduction number (*R*_*0*_; see Box 1) demonstrated that spill‐over from animals is indeed maintaining human schistosomiasis in China, and in the absence of such zoonotic reservoirs, human transmission could be interrupted (Rudge et al. 2013). However, investigation of the relative contribution of each host species demonstrated that it was not only the previously implicated bovines responsible for such zoonotic transmission, but that in hilly regions where cattle are now rare, various rodent species are responsible for maintenance of transmission and spill‐over to humans (Wang et al. 2006; Lu et al. 2009, 2010a,b, 2011; Rudge et al. 2009, 2013). These results also showed that, as a zoonosis, *S. japonicum* could be classified as either a spill‐over pathogen, an apparent multihost pathogen or a true multihost pathogen (Box 1), depending on the ecological location and scale at which the multihost parasite community is observed, all of which may have implications for any subsequent control measures applied. This work also highlighted that estimates of relative host species contributions to overall transmission may be biased by differences in the ease with which they can be captured and sampled; species with low capture probabilities might be underrepresented, such that their role in transmission is underestimated (Rudge et al. 2013; Streicker et al. 2013). In the case of *S. japonicum* in China, there had been no previous sampling recorded from rodent wildlife populations and hence this important potential reservoir host had been ignored. Even if additional zoonotic control measures such as the removal or vaccination of water buffalo were applied, these may not be sufficient to interrupt transmission, or even achieve sustainable control, if rodents subsequently maintain transmission (Rudge et al. 2013). Similar challenges occur in the Philippines, where parasitological and molecular analyses have revealed that dogs are partially responsible for maintaining *S. japonicum* transmission and human infections (Rudge et al. 2008).

A conceptual framework for understanding host species contributions to parasite transmission in a given ecological setting may be helpful. As exemplified above, a parasite's fitness can be measured by its basic reproduction number, R_0_, the number of new infections (for a microparasite, such as a virus, bacteria or protozoan) or new adult parasites (for a macroparasite, such as a helminth or ectoparasite) arising from a primary infection in a wholly susceptible host population or community (Anderson & May, 1991). In a multihost species context, the overall R_0_ of the parasite (*R*_0,TOT_) within a host community will depend on the separate contribution of each host species (R_0,*i*_); only those species with an R_0,*i*_ > 1 will be capable of independently sustaining transmission in the absence of other host species (Fenton and Pedersen 2005; Funk et al. 2013; Fenton et al. 2015). Such a theoretical framework was applied by Rudge et al. (2013) on the zoonotic *S. japonicum*, which allowed the different host species contributions to *R*_0,TOT_ to be quantified using relatively straightforward parasitological data, and important conclusions about transmission and the likely effects of control measures to be made.

The underlying drivers of heterogeneity among host species in how they contribute to transmission, and evolutionary selective forces, also influence optimal control of multihost parasites. Again using mathematical simulations, Streicker and colleagues compared the efficacy of treating infected vs. random individuals under contrasting key host types, and demonstrated very different control implications under each scenario (Streicker et al. 2013). If key hosts arise through high abundance, it is potentially worthwhile making the effort (if possible) to identify and remove only infected key host individuals. In contrast, when key hosts are rare but commonly infected, there may be few gains by identifying infected individuals, and so treating (or in certain cases culling) all individuals regardless of infection status is a more practical approach. Hence, the added diagnostic costs of test‐and‐treat or test‐and‐cull programs, as used to control bovine tuberculosis in African buffalo for example (Jolles et al. 2005), might yield only trivial gains over untargeted control for certain drivers of host heterogeneity. In such circumstances, prioritizing maximization of treatment rates over accurate diagnosis may be optimal, while test‐and‐treat strategies may be highly fruitful in others zoonotic scenarios. In Uganda, for instance, where cattle are the major reservoir of Human African Trypanosomiasis caused by *Trypanosoma brucei rhodesiense,* treatment of all cattle has been demonstrated to be a more cost effective option than a test‐and‐treat approach and is combined by selective spraying of cattle with insecticide thereby reducing both the reservoir of human infection and the vector population (Welburn et al. 2015).

## Animal reservoirs and host switching in response to changing selective pressures

Host switches, the process by which a parasite successfully jumps from one host species to another (Box 1), are thought to have been a major process in the evolution of many zoonotic disease systems. In general, however, very little is known about the ecology and even less the evolution of infectious agents of wildlife, livestock and even companion animals relative to that of humans (Tomley and Shirley 2009), and there are several examples where enzootic viruses of animals (e.g. SARS coronavirus, hantaviruses, Ebola and Marburg viruses, Nipah virus, Hendra virus and human immunodeficiency viruses) were completely unknown until they switched hosts and caused disease in humans (Parrish et al. 2008). The evolutionary potential of a parasite will affect its ability to infect a new host species (Cleaveland et al. 2001; Antia et al. 2003) and disease management methods such as chemotherapy may provide sufficient selection pressure to drive host switching by parasites (Fig. 1). Pathogens with high mutation rates, antigenic diversity and short‐generation times, for example, may rapidly adapt to new host species (Whitlock 1996; Gupta et al. 1998; Woolhouse et al. 2002), and evidence suggests that RNA viruses are the most likely group of infectious agents to host switch and establish in humans, (Woolhouse et al. 2001). If selective pressures are sufficiently strong, however, even organisms with very long generations times, such as many helminth species, have high potential for evolutionary change and host switching (Woolhouse and Webster 2000; Woolhouse et al. 2002). Epidemiological features of disease, routes or modes of transmission, pathogen virulence evolution and host susceptibility may all influence a parasite's ability to switch host species.

More cases of potential reservoir hosts and host switching are likely to be detected as improvements in molecular typing become available. An important example is the Guinea worm *Dracunculus medinensis*, a parasitic worm transmitted by infected copepods consumed when people drink contaminated water. Guinea worm (Dracunculiasis) is currently targeted for global eradication via the Guinea Worm Eradication Program (GWEP), through surveillance, case containment, improved access to clean drinking water, copepod control, provision of water filters, education and behaviour change. In 2013, there were only 542 cases reported in four counties (Chad, Ethiopia, Mali and South Sudan), and by 2014, only 126, mainly in South Sudan (Biswas et al. 2013). However, Dracunculiasis was rediscovered in Chad in 2010 after an apparent absence of 10 years when no human cases were reported (Eberhard et al. 2014). Furthermore, the epidemiologic pattern of the re‐emerged disease in Chad is unlike that seen previously in Chad or other endemic countries, including those now certified as free of transmission. Notably large numbers of dogs were found to be infected in Chad. Molecular sequencing, using both COX‐1 barcoding and whole‐genome sequencing (WGS) indicated these infections, human and dog alike, were all caused by *D. medinensis*. It thus appears that the infection in dogs is serving as the major driving force sustaining transmission in Chad, with potentially an aberrant life cycle involving a paratenic host (Box 1) involved in transmission to both humans and dogs (Eberhard et al. 2014). A life cycle which involves a paratenic host has been reported from *Dracunculus insignis*, a parasite of racoons in North America, and in *D. insignis* from ferrets in laboratory studies (Crichton and Beverly‐Burton 1977; Eberhard et al. 2014). It is postulated that dogs have become infected by eating fish entrails which have been discarded when fish are dried for later human consumption, with fish acquiring infection from copepods and maintaining viable *D. medinensis* larvae. Such observations suggest that, rather than being eradicated in Chad, Guinea Worm may have host switched from predominantly infecting humans to being a canine zoonotic infection (although due to a scarcity of rigorous sampling of the canine populations previously, the possibility that they have always harboured Guinea worm infections cannot be ruled out). *Dracunculus medinensis* has also been occasionally reported from cats, horses, cows, wolves, leopards, monkeys and baboons (Genis 1972). Further research into and monitoring of dogs, and ideally also cats and wildlife, as viable hosts for this parasite, combined with further WGS to elucidate potential selective pressures and host shifts, are now required. Of concern is that the number of cases of *D. medinensis* in dogs in Chad continues to escalate (Center 2015). Guinea worm was a parasite targeted for global eradication in part because it was believed to be a human parasite with no zoonotic reservoir. More broadly, this Guinea worm example highlights a rarely considered, general challenge for disease control programmes, that control efforts in one target host species (e.g. humans or livestock) may provide a selective pressure for parasites to expand or shift their zoonotic host range, thereby enabling them to be maintained in new reservoir host species (Fig. 1).

There is also evidence from another helminth, *Ancylostoma ceylanicum*, a hookworm of canids and felids in Asia, of transmission from these animals to humans, even in the era of human‐targeted mass drug administration against hookworm and other soil‐transmitted helminths. In a recent study which examined both humans and dogs in a rural Cambodian village, over 57% of the human population was infected with hookworms and of those, around half harboured *A. ceylanicum*. Over 90% of the dogs examined were also infected with *A. ceylanicum*. Furthermore, characterization of the COX‐1 gene subdivided the *A. ceylanicum* isolates into two groups, one containing isolates from humans only and the other a mix of isolates from humans and dogs. The authors proposed that human‐targeted chemotherapy alone, in the absence of concurrent animal health programs, may have facilitated a host switch and establishment of *A. ceylanicum* infections in humans (Inpankaew et al. 2014).

Among schistosomes, while the Asian species *S. japonicum* and *S. mekongi* are accepted as zoonotic, there is evidence that human *S. mansoni* has recently switched to being maintained in nonhuman primates in areas of Africa, such as the National Parks in East Africa, where human hosts are no longer available (Muller‐Graf et al. 1997). Similarly, following extensive human‐targeted control programmes, *S. mansoni* appears to have switched back towards a murid definitive host (*Rattus rattus*) in Guadeloupe, the French West Indies (Theron 1984; Theron et al. 1992). A study in Kenya, using COX‐1 barcoding, revealed that 6% of the rodents and insectivores sampled were infected with a range schistosome species traditionally believed to the livestock or human‐specific, including *S. bovis* and *S. mansoni,* in addition to their rodent schistosome species *S. rodhaini* (Hanelt et al. 2010). Although the prevalence of *S. mansoni* infection in these reservoir populations was low (1·5%), as with *S. japonicum* in rodents across China, these species have potentially vast population sizes, giving them potential to be ‘super‐abundant’ key hosts, with implications for optimizing control measures (Streicker et al. 2013). The potential role of these highly abundant small wild animals in host switches and subsequent ongoing parasite transmission, particularly perhaps following large‐scale human chemotherapy programmes, warrants further research.

## Mixed species co‐infections and introgressions

Multiparasite systems are evolutionarily as well as ecologically dynamic. Hence, effective disease control of NZDS must consider not only how best to deal with reservoir hosts and host switching, but also the possibility of novel parasites evolving and establishing. Polyparasitism, that is co‐infection with more than one parasite species, is the norm in animal populations, including humans, and particularly in the developing world (Mupfasoni et al. 2009). For instance, across much of sub‐Saharan Africa, humans can be co‐infected with *S. mansoni* and *Schistosoma haematobium* (Webster et al. 2012; Garba et al. 2013), their domestic livestock co‐infected with *S. bovis, S. curassoni* and/or *S. mattheei* (Rollinson et al. 1990), and rodent wildlife co‐infected with *S. mansoni* and *S. rodhaini* (Hanelt et al. 2010). Through synergistic or antagonistic (including competitive exclusion) interactions among parasites, co‐infection may influence parasite establishment, growth, maturation, reproductive success and drug efficacy (Norton et al. 2008; Webster et al. 2008). Additionally, co‐infection by species belonging to the same genus can allow heterospecific (between species) pairings, resulting in either parthenogenesis (asexual reproduction where eggs occur without fertilization) or introgression (the introduction of genes from one species into that of another) and the production of hybrid offspring (Box 1: Fig. 1). Introgression, also known as introgressive hybridization, in genetics is the movement of a gene (gene flow) from one species into the gene pool of another by the repeated backcrossing of an interspecific hybrid with one of its parent species (Box 1). Introgression is an important source of genetic variation in natural populations and may contribute to adaptation and even adaptive radiation (Mavarez et al. 2006; Pardo‐Diaz et al. 2012). Introgression differs from simple hybridization. Introgression results in a complex mixture of parental genes, while simple hybridization results in a more uniform mixture, which in the first generation will be an even mix of two parental species. Genetic exchange among parasite species has potential implications for heterosis (hybrid vigour), the emergence and spread of novel and virulent strains, differential sensitivity to chemotherapy, increased host range and adaptation to new ecological niches that may provide a selective advantage. While hybrid offspring are usually sterile, recent molecular developments have revealed instances of fertile hybridizations, or introgressions, in plants, animals and occasionally parasites (Detwiler and Criscione 2010; King et al. 2015), which could have major impacts on species diversification and host range (Consortium* 2012), infection persistence, drug resistance and clinical outcomes, and poses further challenges for effective control. In addition, pathogen evolution through introgression may greatly affect the likelihood of disease emergence by increasing the pathogen's basic reproduction number (*R*_0_). For example, avian influenza has emerged several times in human populations since 1997. Typically, limited human‐to‐human transmission exists, so that although the avian reservoir and susceptible human populations are large, outbreaks are rare and isolated (Fenton and Pedersen 2005). Only through recombination between strains and acquisition of human‐specific respiratory epithelium receptors could the virus evolve sufficient transmissibility to be sustained in the human population, which poses the greatest risk for pandemics (Webby and Webster 2003). These genetic changes could shift avian flu from being a spill‐over to becoming a true multihost parasite, which would have serious implications for human health.

Evidence for hybridizations and introgressions between parasite species is gathering, at least in part in line with improvements in molecular diagnostics and genome sequencing of these organisms. Examples include several causative agents of important NZDS caused by helminths, such as *Schistosoma, Fasciola, Ascaris* and *Trichinella* (Schistosoma, Fasciola, Ascaris, and Trichinella; Criscione et al. 2007; Dunams‐Morel et al. 2012; Le et al. 2008; Webster et al. 2013a), protozoa, such as *Plasmodium, Leishmania, Toxoplasma* and *Trypanosoma* (Akopyants et al. 2009; Gaunt et al. 2003; Grigg et al. 2001; Machado and Ayala 2001; Ramiro et al. 2015; Rogers et al. 2014; Sturm et al. 2003), as well as their vectors (King et al. 2015). For instance, research on *A. lumbricoides* and *A. suum* collected from sympatric human and pig populations in both Guatemala and China found that between 4 and 7% of the roundworms sampled were hybrids. Similarly, Yamane and colleagues examined COX‐1 and two nuclear genes in tapeworms obtained from humans in the Tibetan Plateau of Sichuan, China, where *Taenia saginata*, a parasite of cattle, and *T. asiatica*, a parasite of both humans and pigs are sympatrically endemic (Yamane et al. 2012). Phylogenetic analyses revealed that some adult worms showed nuclear‐mitochondrial discordance, suggesting they originated from hybridization, and due to the nature of the discordance, that reciprocal hybridization between *T. saginata* and *T. asiatica* could occur. The authors concluded that although self‐fertilization, and hence, inbreeding are thought to be the main reproductive mode of *Taenia*, heterozygosity at nuclear loci suggested ongoing hybridization between *T. asiatica* and *T. saginata* in many co‐endemic areas. Furthermore, subsequent phylogenetic analyses by the same authors, using partial sequences of the DNA polymerase delta (pold) gene, revealed that adult worms previously identified as *T. asiatica* using mitochondrial DNA, were homozygous for an allele that differed from a *T. saginata* allele by a single nucleotide substitution. Such results indicate that most adult worms previously assumed to be *T. asiatica* were in fact not actually ‘pure *T. asiatica’* but instead originated from the hybridization of between *T. saginata* and *T. asiatica* (Yamane et al. 2013).

There is also an increasing literature on introgressions in the protozoal NTDs leishmaniosis and trypanosomiasis (Miles et al. 2009; Messenger et al. 2012, 2015). Approximately 30 000 people in 36 countries of sub‐Saharan Africa suffer from human African trypanosomiasis (HAT), which is caused by either *Trypanosoma brucei gambiense* or *Trypanosoma brucei rhodesiense*. The other human form of human trypanosomiasis, Chagas disease, has been classed as the most important vector‐borne infection in Latin America, affecting an estimated 7–8 million individuals with around 21 000 deaths per year (WHO 2014). *Leishmania* parasites give rise to a spectrum of diseases ranging from the dermal lesions of cutaneous leishmaniasis to the organ failure of visceral leishmaniasis. While there are currently no known reservoir hosts of visceral leishmaniasis in India, Nepal and Bangladesh, where the most serious mortality occurs, both *Leishmania* spp. and *Trypanosoma* spp. are parasites with arrange of major potential zoonotic reservoirs, encompassing both domestic livestock and wildlife (Table 1). For example, at least two of the six major lineages (distant typing units) of *T. cruzi* (III & IV) are now believed to have arisen by hybridization, despite their predominant mode of asexual reproduction, with lineages differing in their biology, drug sensitivity and host range (Miles et al. 2009). Furthermore, evidence from laboratory crosses of the HAT infectious agent *T.b. rhodiesenses* and the morphologically indistinguishable nonhuman infective subspecies *T.b. brucei*, involving sexual reproduction within the tsetse fly vector, demonstrated that the Serum Resistance Associated (*SRA*) virulence gene, which is the single gene that governs human infectivity in *T.b. rhodesiense,* can be transferred by genetic exchange to *T.b.brucei strains*, thus creating new genotypes of potentially human infective parasites (Gibson et al. 2015). The authors thus concluded that new strains of the human pathogen *T.b. rhodiense* are likely to be being generated continuously by recombination with the much larger pool of animal‐infective trypanosomes and that such novel recombinants present a risk for future outbreaks of HAT.

Similarly, while asexual reproduction through clonal propagation is thought to be the major reproductive mode for *Leishmania*, a sexual cycle has been detected experimentally within the sand fly vector, and sexual recombination under a range of endemic field conditions has long been suspected, based on hybrid marker profiles (Kelly et al. 1991; Rogers et al. 2014). For example, intraspecific hybridization events between *L. donovani* clones in Ethiopia (Gelanew et al. 2014) and *L. infantum* clones in Tunisia (Chargui et al. 2009), as well as interspecifically, for example between the closely related *L. braziliensis* and *L. guyanensis* in Venezuala (Delgado et al. 1997) and the much more widely divergent *L. infantum* and *L. major* where hybrids were isolated from immune‐compromised patients in Portugal (Ravel et al. 2006). The potentially important evolutionary and epidemiological consequences of such events are highlighted by the observation that *Leishmania infantum:L. major* hybrids possess enhanced transmission potential, since unlike either of their parental single species, hybrid offspring are able to infect an additional sandfly vector species, the geographically broader ranging *Phlebotomus papatasi* (Volf et al. 2007).

Another topical example of the potential for introgression to affect disease transmission through changes in host range is provided by *S. haematobium*, the causative agent of urogenital schistosomiasis. *Schistosoma haematobium* was believed to be an exclusively human‐specific parasite and the risk of zoonotic transmission in Africa was deemed safe to ignore. However, as early as the 1940s there were suggestions of potential hybridizations involving different *Schistosoma* species, initial based on variable egg morphologies, but later, from the 1990s, using molecular tools such as enzyme electrophoresis (reviewed in Leger and Webster in press). More recently ITS1 + 2 and COX‐1 studies of schistosome miracidial larvae collected and successfully hatched from the stool and urine of Senegalese school children confirmed bidirectional hybridization between *S. haematobium* of humans and *S. bovis* of livestock (Huyse et al. 2009), and between *S. haematobium* of humans and *S. curassoni* of livestock (Webster et al. 2013a). Similar studies on infected snail intermediate hosts in Kenya, using microsatellite markers, rDNA and mtDNA, also observed hybrid cercariae between *S. mansoni* of humans and its sister species, *S. rodhaini*, of rodents (Steinauer et al. 2008). Furthermore, these hybrids were demonstrated to produce viable offspring through first and successive generation backcrosses with *S. mansoni*. In this case, however, the direction of introgression appeared highly asymmetric, with unidirectional gene flow from the rodent parasite, *S. rodhaini*, to the human parasite, *S. mansoni* (Steinauer et al. 2008). Recent evidence from eggs and miracidia from infected humans in Senegal have also detected potential introgressions between the more phylogenetically distant *S. haematobium* and *S. mansoni*, pairings between these two species that was previously believed to result in unviable eggs exclusively through parthenogenesis (Huyse et al. 2013). Such evolutionary changes in these parasite populations are likely to have major implications in light of the current global push for human chemotherapy control programmes to shift from controlling morbidity to halting transmission. Further empirical work is now necessary to confirm the epidemiological importance of these hybridization events, for example to monitor how their prevalence and genetic composition changes over time, and in response to varying environmental conditions and chemotherapy. Phylogenetic studies may identify where and how these hybrids arise, and help predict where they may arise in the future. A particularly pertinent question is whether the evolution and expansion of zoonotic hybrid schistosomes in Africa is a recent phenomenon in response to new anthropogenic changes and pressures, in particular the wide‐scale treatment of human populations with praziquantel, or simply recently detected through improvements in molecular diagnostics. Evidence in favour of the former may be provided by the observation that hybrids have not been detected across all countries and regions examined to date. For instance, no hybrid schistosomes have been detected in Zanzibar, despite extensive molecular analytical studies being performed. There is therefore a need to understand what drives the occurrence of hybrids in some locations and not others. In the context of current control efforts, there is a need to determine whether the efficacy of praziquantel, the only currently used antischistosome drug, differs for hybrid parasites, and whether this could partly or wholly explain recent poor responses to preventative chemotherapy in areas where hybrids are prevalent. It is also important to understand whether hybrid infections present a differential human (and animal) morbidity profile, as a result of, for example, either hybrid vigour and/or the hybridization of intestinal with urogenital schistosome species (Koukounari et al. 2010; Gouvras et al. 2013)). Such differential morbidity in hybrid infections may have major implications for current methods of monitoring human morbidity levels and control programme efficacy. Finally, how such introgression may alter host range, and hence, transmission potential is perhaps the most pressing area for future research. Many schistosome species infecting livestock have a broader geographical range beyond sub‐Saharan Africa, where compatible snail intermediate hosts are present. Novel zoonotic hybrids may therefore have the potential to become not simply an NTD, but more of a global disease, particularly with global warming, increased human and animal movement and transportation. The cattle schistosome *S. bovis,* for example, has a naturally wide definitive host spectrum and is compatible, at least experimentally, with a wide range of intermediate snail host species (Moné et al. 1999). In Europe, human urogenital schistosomiasis, associated with *S. haematobium,* has previously been detected in Portugal, where this focus disappeared during the 1950s (Fraga de Azevedo et al. 1948). However, freshwater snails of the species *Bulinus contortus*,* B*. *truncatus* and *Planorbarius metidjensis*, which are recognized intermediate hosts for *S. haematobium*, have been found in Portugal (de Azevedo 1969), Spain (Silva Oliveira et al. 1974; Perez‐Quintero et al. 2004) and Corsica (Gretillat 1963; Doby et al. 1966). These findings suggest that urogenital schistosomiasis could re‐emerge and establish in southern Europe, if these molluscs become infected. Furthermore, introgressed hybrids between human *S. heamatobium* and livestock *S. bovis* have recently been identified, with potentially substantial ongoing transmission among both local residents and tourists, in Corsica, Europe (Berry et al. 2014; ECDC 2014; Boissier et al. 2015).

For all recently identified parasite introgressions, we now need to identify in which host species the actual hybridization events actually occur. Furthermore, if such hybridizations are maintained through successive generations, we need to identify and distinguish between potential spill‐over and key hosts that are driving ongoing transmission. For example, hybrids between human with animal *Schistosoma* spp. have, to date, only been identified in humans and snail intermediate hosts, but future work must assess the potential role of both livestock and wildlife. Identifying parasite transmission events in livestock and wildlife populations is notoriously challenging. Nevertheless, future quantitative and qualitative data sets and multihost R_0_ framework analyses on the occurrence and consequences of hybridization and introgression would enable exploration of the possible movement of any emerging potential hybrid through that parameter space as it evolves, for instance, towards generalist or specialist on the available host species.

## Potential uses of animal reservoirs in human disease control

The NZDs, particularly those that infect livestock, pose additional indirect risks to the health of human populations through their impact on livelihoods, poverty and potentially nutrition. However, the role of zoonotic reservoirs in disease control need not be all entirely negative, and their advantages versus disadvantages must be fully understood. One potential upside is that disease control in a key reservoir species, for example domestic animals, may be more practical than in humans. New vaccines are often easier and faster to develop for animal than for human use, particularly in terms of safety and clinical trial regulations. Moreover, targeting the animal reservoir can also limit the number of new human cases and their associated morbidity, which is particularly relevant for diseases with a high mortality rate, or for which treatment itself carries risks, such as trypanosomiasis. Spraying cows with insecticide to control tsetse flies has, for instance, been shown to be effective for the control of *T. rhodesiense* –associated human African trypanosomiasis (Welburn et al. 2001; Hargrove et al. 2012; Muhanguzi et al. 2014). Furthermore a combined approach of chemotherapy in cattle as well as spraying cattle with insecticide has been shown to be particularly effective in Uganda, where cattle movement threatens the spread of *T. rhodesiense* into areas endemic for the chronic form of the disease in the north‐western parts of the country (Hargrove et al. 2012) (Hargrove et al. 2012).

Alternative host species may also play a role in reducing infection risk through the ‘dilution effect’ (Keesing et al. 2006). In this scenario, host species that had low capacity for onward transmission may ‘absorb’ or disrupt infectious contacts, thereby reducing the force of infection on a target host species. This has been classically thought of in terms of tick‐borne diseases (e.g. Lyme disease), where infection opportunities are lost through ‘wasted bites’ by ticks on noncompetent host species. Although there is considerable debate about the generality of this phenomenon (Randolph and Dobson 2012; Wood et al. 2014) and the mechanisms may be complex and hard to disentangle (Salkeld et al. 2013; Strauss et al. 2015), evidence is accumulating to support it in a number of systems (Civetello et al. 2014) suggesting that increased biodiversity can be beneficial in terms of reducing infection risk, at least in some circumstances.

Animal reservoirs may also have a positive role in the context of reducing the risk of drug resistance evolving. It is suspected that one reason praziquantel resistance has not established in China, despite extensive preventive chemotherapy for schistosomiasis, is the existence of animal host reservoirs, and hence large *Refugia* (Box 1) for parasites. Should there also be large domestic and/or wild animal reservoirs for the African human schistosomes (a possibility that requires further investigation), this could actually mitigate against the establishment or spread of praziquantel resistance in these areas. The relative importance of nonhuman hosts as both sources of infection, and conversely as *Refugia*, will, however, depend on whether the parasite population is panmictic or whether different parasite strains circulate independently in different host species, that is whether parasites are partial or wholly covert host specialists. Drug selection pressures are also suspected to differ between parasite species, with potential implications both for and against the potential emergence of praziquantel resistance (Webster et al. 2008).

While zoonotic infections may be harder to eliminate due to the presence of animal reservoirs driving transmission, other processes may select for reduced human infection. If parasite strains vary in host preference, selection imposed by drug treatment in humans may lead to a shift in host preference, favouring strains that prefer nonhuman hosts. Observations from Guadeloupe suggest such a process may have occurred in *S. mansoni* there. Phenotypic and genetic studies suggest the recent adaptation of *S. mansoni* (back) to its rodent host has resulted in a phenotypic change (the time of cercarial emergence), that may decrease the risk of human infection (Theron 1984; Theron et al. 1992). Even in a country as large as China, there is evidence that some genetic and phenotypic isolation may be occurring, with a more rodent‐philic *S. japonicum* being selected (Wang et al. 2006; Lu et al. 2009, 2010a,b, 2011; Rudge et al. 2009, 2013). Further, it appears *S. japonicum* in China may show local adaptation to the use of different definitive host reservoirs; in hilly regions where nocturnal rodents predominantly maintain transmission, cercariae are being shed from *Oncomelanaia* snails in the late afternoons and evening, while in lowland habitats where bovines drive transmission, early morning shedding occurs, which coincides with the timing of peak bovine water contact (Lu et al. 2009, 2011). Such patterns may be the consequence of intense human chemotherapeutic pressure in China, selecting increasingly zoophilic parasites. This raises the question of what may happen when drug‐imposed selective pressure ceases. The answer may depend on the extent of parasite divergence by the time this pressure is removed. If no genetic isolation has evolved between parasite genotypes circulating in humans and animals, one would predict continued transmission to humans and re‐establishment as a human disease. This situation may be relevant currently for *S. japonicum* in the Philippines (Rudge et al. 2008) and China (Rudge et al. 2009), where, although adaptation towards different host species does appear to be occurring, species‐specific private alleles (alleles found only in a single population) appear to be circulating across both human and animal hosts. However, if complete or near complete genetic isolation between parasites in animal and human hosts occurs, one could predict the evolution of an exclusively zoophilic parasite. Findings from Taiwan may indicate such an outcome, where although *S. japonicum* continues to infect a broad range of animals, it now appears unable to infect humans (Fan 2006).

## Ecological and evolutionary applications – optimizing disease control for NZDs

We live in a time where disease ‘elimination’ and even ‘eradication’ have been proposed as Millennium Development Goals (WHO 2012b). These goals are difficult to achieve for any infectious disease, as reflected by the fact that only one human and one animal pathogen have been globally eradicated to date (smallpox and rinderpest; (Klepac et al. 2013). The challenges of elimination are magnified for zoonotic parasites (Taylor et al. 2001). To eliminate zoonotic infections, one must not only eliminate infection in the human population, but also prevent or eliminate transmission from animal reservoirs. Moreover, parasite evolution occurs across multiple hosts. This added ecological and evolutionary complexity has key implications for designing effective disease control strategies. For example, infection control may need to target multiple host species, or block transmission pathways between animals and humans. Moreover, the set of tools required for control are likely to be more diverse for zoonoses. These parasites may show genetic diversity across different host species, such that a vaccine or drug effective in one host species, may not be in another. Moreover, drug treatment of animal reservoirs, even with different drugs to those used in humans, may lead to the development of cross‐resistance, rendering human drug treatment less effective (Maia et al. 2013). Simply expanding control measures used in humans to animal reservoirs may often not be the best solution (Maia et al. 2013). Evidence‐based strategies that exploit a sound understanding of each NZD's ecology and ongoing evolution are likely to be more fruitful. While elimination of most NTDs with zoonotic reservoirs may be aspirational rather than realistic at present, good progress continues to be made on the control of several important NTDs, and mechanisms are now in place to bring together the critical scientific expertise and political will to significantly improve control of NZDs.

Typically, zoonotic diseases are managed, if at all, through separate interventions across the human and animal health sectors. However, there is a clear public health imperative for approaches that take into account the full One Health perspective, by understanding the transmission ecology and evolution of NZDs across all hosts they infect, both animal and human. Each NZD is likely to be different, with optimal control depending on (i) exactly which animals contribute to transmission and in what way; (ii) the particular routes of animal‒human transmission; and (iii) known evolutionary changes occurring in the parasite that may affect for example parasite population genetics and host range or response to control measures. Given the global health agenda and widespread scaling up of NTD control programs (Boatin et al. 2012; Lustigman et al. 2012), studies that elucidate the complex transmission ecology of NZDs are especially timely. Evolutionary theory has a role to play in informing NZD control programme design. Perhaps its most important role will be to help predict how treatment imposed selection may drive evolutionary change in NZDs, thereby facilitating the design of effective monitoring processes, capable of detecting such changes as they occur.

Successful NZD control is likely to involve state‐of‐the‐art approaches for diagnosis and surveillance across humans and animal hosts, to provide a network of global intelligence on their distribution, potential for spread, and disease burden. Control of NZDs calls for integrated collaboration among governments and nongovernmental organizations engaged at the human–animal–ecosystems interface, between researchers from medical, evolutionary, ecological and social fields, and strengthening information exchange across relevant sectors in affected countries, particularly agriculture and human health.

Ultimately, to impact policy for any given NZD, we first need a sound empirical evidence base concerning multihost transmission ecology, behaviour, morbidity, drug efficacy and potential evolutionary changes such as introgression occurring in different host species. This empirical evidence then needs to be allied with robust theoretical and computational methods to understand the drivers of transmission, and explore alternative disease management scenarios. We make the following specific recommendations on what is needed to successfully tackle both ancient and emerging NZDs in the future:

1. We need a detailed empirical understanding of the biology and relative importance of each key host species. This involves not only understanding how to reliably identify key host species within multihost parasite systems, but also the magnitude and precise nature of their contribution to transmission, which may involve a variety of processes (Fenton and Pedersen 2005).
2. We need improved cutting‐edge genetic and genomic tools to differentiate infectious agents, across both species and strain level. Such tools will enable researchers to track the gene flow of key infectious agents, for instance between wildlife, domestic animals and humans, as well as reveal, for instance, novel host switching and introgression events that are currently missed with existing tools unless they involve recombination between very different genetic backgrounds.
3. We need the development of methods for high quality behavioural tracking of hosts that can characterize in unprecedented detail their social networks and the barriers and conduits for spread within and between species.
4. The application of novel mathematical and statistical approaches from the fields of statistical, computer science and network analysis will be important in order to model gene flow across such multihost parasite transmission networks and to provide a quantitative understanding of transmission ecology for multihost parasites. Furthermore, molecular studies combined with mathematical modelling using an R_0_ framework will be critical to identify key hosts and hence identify targets for control. Such modelling will require empirical evidence on factors such as differential pathogen susceptibility to control measures (e.g. drug susceptibility), patterns of interaction between humans, livestock and wildlife, and integration with data collected from other sources such as livestock numbers. Ideally we should also look to extend these frameworks to explore potential evolutionary responses of the parasites to imposed control measures, for example to understand the likely impacts of different control measures on patterns of parasite evolution including drug resistance and hybridization.
5. Finally, if we are to fully understand the impact of NZDs on human disease as well as how relationships between the causative parasites and their hosts may be changing, we need to stress the importance to policy makers of incorporating each of the above into expanded monitoring and evaluation components inherent in all human and animal disease control efforts.

The notable parasitologist Claude Combes stated ‘Tell me what parasites you have and I'll tell you who you are’ (Combes 2005). However, as our global environment changes, including changes in livestock practices and the selective pressures imposed by drugs and vaccinations, combined with rapidly developing molecular tools allowing finer scale identification of species and species complexes, it is possible that additional parasites may ‘emerge’, and our disease monitoring and prevention efforts will have to continuously adapt if we are to effectively control them.
